# Cluster randomized trial of a multilevel evidence-based quality improvement approach to tailoring VA Patient Aligned Care Teams to the needs of women Veterans

**DOI:** 10.1186/s13012-016-0461-z

**Published:** 2016-07-19

**Authors:** Elizabeth M. Yano, Jill E. Darling, Alison B. Hamilton, Ismelda Canelo, Emmeline Chuang, Lisa S. Meredith, Lisa V. Rubenstein

**Affiliations:** 1 VA HSR&D Center for the Study of Healthcare Innovation, Implementation and Policy, VA Greater Los Angeles Healthcare System, 16111 Plummer Street, Building 25 (Mailcode 152), Sepulveda, CA 91343 USA; 2 Department of Health Policy & Management, UCLA Fielding School of Public Health, 650 Charles E. Young Drive South, Center for Health Sciences, Los Angeles, CA 90095-1772 USA; 3 Department of Psychiatry and Biobehavioral Sciences, UCLA Geffen School of Medicine, 760 Westwood Plaza, Los Angeles, CA 90095 USA; 4 RAND Health, 1776 Main Street, Santa Monica, CA 90401-3208 USA; 5 Department of Medicine, VA Greater Los Angeles Healthcare System, 11301 Wilshire Blvd., Los Angeles, CA 90073 USA; 6 Department of Medicine, UCLA Geffen School of Medicine, 611 Charles E. Young Drive East, Los Angeles, CA 90095 USA; 7 Dornsife Center for Economic and Social Research, University of Southern California, 635 Downey Way, Los Angeles, CA 90089 USA

**Keywords:** Implementation, Evidence-based quality improvement, Patient-centered medical home, Women’s health, Veterans

## Abstract

**Background:**

The Veterans Health Administration (VA) has undertaken a major initiative to transform care through implementation of Patient Aligned Care Teams (PACTs). Based on the patient-centered medical home (PCMH) concept, PACT aims to improve access, continuity, coordination, and comprehensiveness using team-based care that is patient-driven and patient-centered. However, how VA should adapt PACT to meet the needs of special populations, such as women Veterans (WVs), was not considered in initial implementation guidance. WVs’ numerical minority in VA healthcare settings (approximately 7–8 % of users) creates logistical challenges to delivering gender-sensitive comprehensive care. The main goal of this study is to test an evidence-based quality improvement approach (EBQI) to tailoring PACT to meet the needs of WVs, incorporating comprehensive primary care services and gender-specific care in gender-sensitive environments, thereby accelerating achievement of PACT tenets for women (Women’s Health (WH)-PACT).

**Methods/design:**

EBQI is a systematic approach to developing a multilevel research-clinical partnership that engages senior organizational leaders and local quality improvement (QI) teams in adapting and implementing new care models in the context of prior evidence and local practice conditions, with researchers providing technical support, formative feedback, and practice facilitation. In a 12-site cluster randomized trial, we will evaluate WH-PACT model achievement using patient, provider, staff, and practice surveys, in addition to analyses of secondary administrative and chart-based data. We will explore impacts of receipt of WH-PACT care on quality of chronic disease care and prevention, health status, patient satisfaction and experience of care, provider experience, utilization, and costs. Using mixed methods, we will assess pre-post practice contexts; document EBQI activities undertaken in participating facilities and their relationship to provider/staff and team actions/attitudes; document WH-PACT implementation; and examine barriers/facilitators to EBQI-supported WH-PACT implementation through a combination of semi-structured interviews and monthly formative progress narratives and administrative data.

**Discussion:**

Lack of gender-sensitive comprehensive care has demonstrated consequences for the technical quality and ratings of care among WVs and may contribute to decisions to continue use or seek care elsewhere under the US Affordable Care Act. We hypothesize that tailoring PACT implementation through EBQI may improve the experience and quality of care at many levels.

**Trial registration:**

ClinicalTrials.gov, NCT02039856

## Background

Women Veterans’ (WVs) numerical minority in Veterans Health Administration (VA) healthcare settings has created logistical challenges to delivering gender-sensitive comprehensive primary care (PC). Women commonly must access an array of VA and non-VA providers, usually requiring multiple separate visits, to achieve the same basic level of care which male veterans can achieve through a single on-site PC visit [1–3]. The VA has invested in a variety of resources intended to improve WVs’ care, establishing women’s health clinics, designating Women’s Health (WH) PC providers with the requisite training/experience, and contracting with non-VA obstetrics and gynecology services. Despite these investments, WVs’ quality of care in VA continues to lag behind that of male veterans [4]. Contributing to these outcomes is the lack of gender sensitivity prevalent in many VA care settings, which may be linked to low WV retention rates in VA care [5, 6].

The VA has undertaken a major initiative to transform care through mandated implementation of Patient Aligned Care Teams (PACTs), which hold promise for addressing many of the gaps in WVs’ care. The PACT model is based on the concept of patient-centered medical homes (PCMHs), widely endorsed by PC professional societies and shown to improve quality of care and patient, provider, and staff satisfaction, while reducing costs [7, 8]. PACT focuses on development of high-performing “teamlets” comprised of PC providers, nurses, and administrative support who together manage care of a defined panel of patients. These teamlets operate within a larger team that includes, for example, pharmacists, social workers, mental health (MH) providers, and dietitians, and link to specialists and hospital care in their medical “neighborhood.” Through these teams/teamlets, PACT aims to achieve improvements in accessibility, continuity, coordination, and comprehensiveness using team-based care that is patient-driven and patient-centered [9]. These improvements, in turn, should translate into better chronic illness care and prevention and lower costs.

However, how VA should adapt this major reorganization to meet the needs of special populations, such as WVs, is yet to be fully worked out. The PACT model itself does not include specific accommodations for gender-specific care or improved gender sensitivity. Current WV care is also out of step with PACT priorities and emphasis on “one-stop shopping” for care [10]. For example, WVs are more likely to be outsourced to the community for gender-specific services now than they were 10 years ago, and, while the number of women’s clinics is up, over 40 % do not deliver comprehensive PC [3]. Therefore, improving VA PC alone or “beefing up” women’s clinics is unlikely to achieve what an integrated WH-PACT model must reconcile to improve care for WVs and thereby reduce persistent gender disparities in VA care [11, 12].

### Research aims

We propose to use evidence-based quality improvement (EBQI) in the context of the Chronic Care Model to develop and test achievement of WH-PACT in a cluster randomized trial [13, 14]. EBQI is a systematic approach to developing local research-clinical partnerships to produce tailored, evidence-based care models or redesigns [15]. The resulting WH-PACT redesign will make use of local WH resources (e.g., women’s clinics, designated providers), while linking them to the broader PACT initiative and medical center resources. We will also explore the extent to which receiving care that meets WH-PACT tenets translates into higher value (better quality, lower costs) for individual WVs, evaluate local implementation, and develop tools for sustaining and spreading WH-PACT.

Our aims are:

1. *To assess the effectiveness of EBQI for developing a WH-PACT model using a cluster randomized trial design*. WH-PACT model achievement includes (a) *PACT features* (accessible, continuous, coordinated, team-based, patient-driven, and patient-centered), (b) *comprehensive WH care* (PC, gender-specific care, and integrated MH), and (c) *gender-sensitive care delivery*. We will (a) survey providers/staff on achievement of WH-PACT model attributes, (b) survey WV patients on WH-PACT model care experiences, and (c) analyze WH-PACT achievement (e.g., continuity) using secondary data.
2. *To examine impacts of receipt of WH-PACT concordant care on WVs’ outcomes.*
  We will explore impacts on quality of chronic disease care and prevention, health status, utilization and costs.
3. *To evaluate the processes of EBQI-supported WH-PACT implementation*. We will assess pre-post practice contexts; document EBQI activities and their relationship to provider/staff and team actions and attitudes; document WH-PACT implementation; and examine barriers and facilitators to EBQI-supported WH-PACT implementation using mixed methods (e.g., semi-structured interviews, brief progress narratives).
4. *To develop implementation and evaluation tools for use in EBQI-supported WH-PACT model adaptation*, *implementation*, *sustainability*, *and spread to additional VA facilities*.

## Methods/design

### Setting, site selection, trial design, and participants

The VA healthcare system is currently organized into 21 regional Veterans Integrated Service Networks (VISNs), with administrative and clinical authority over VA medical centers (VAMCs) and their affiliated programs in geographically distinct regions of the USA [16].

Study sites are members of the VA Women’s Health Practice-Based Research Network (WH-PBRN), a 37-VAMC network spanning 17 VISNs and comprising over 270 geographically distinct sites of care and nearly 600 designated WH providers [17]. Together, PBRN facilities serve over 100,000 WVs (about one third of WVs seen in VA) and span diverse patient populations. We identified five VISNs with three or more WH-PBRN sites; we excluded one that was already participating in a VISN-wide EBQI stepped-wedge trial. We approached leadership at the other four VISNs and their WH-PBRN member site leads about study participation. All agreed to participate. One VISN dropped out ahead of randomization, which was replaced with a VISN that had two WH-PBRN sites; we then worked with VISN leadership to identify a third non-PBRN VAMC, which subsequently joined the WH-PBRN (since the trial began, the WH-PBRN has expanded to 60 VAMCs in 20 of 21 VISNs). VAMCs participating in the trial span nine states (Connecticut, Illinois, Iowa, Massachusetts, Minnesota, North Dakota, Pennsylvania, West Virginia, and Wisconsin).

**Fig. 1:**
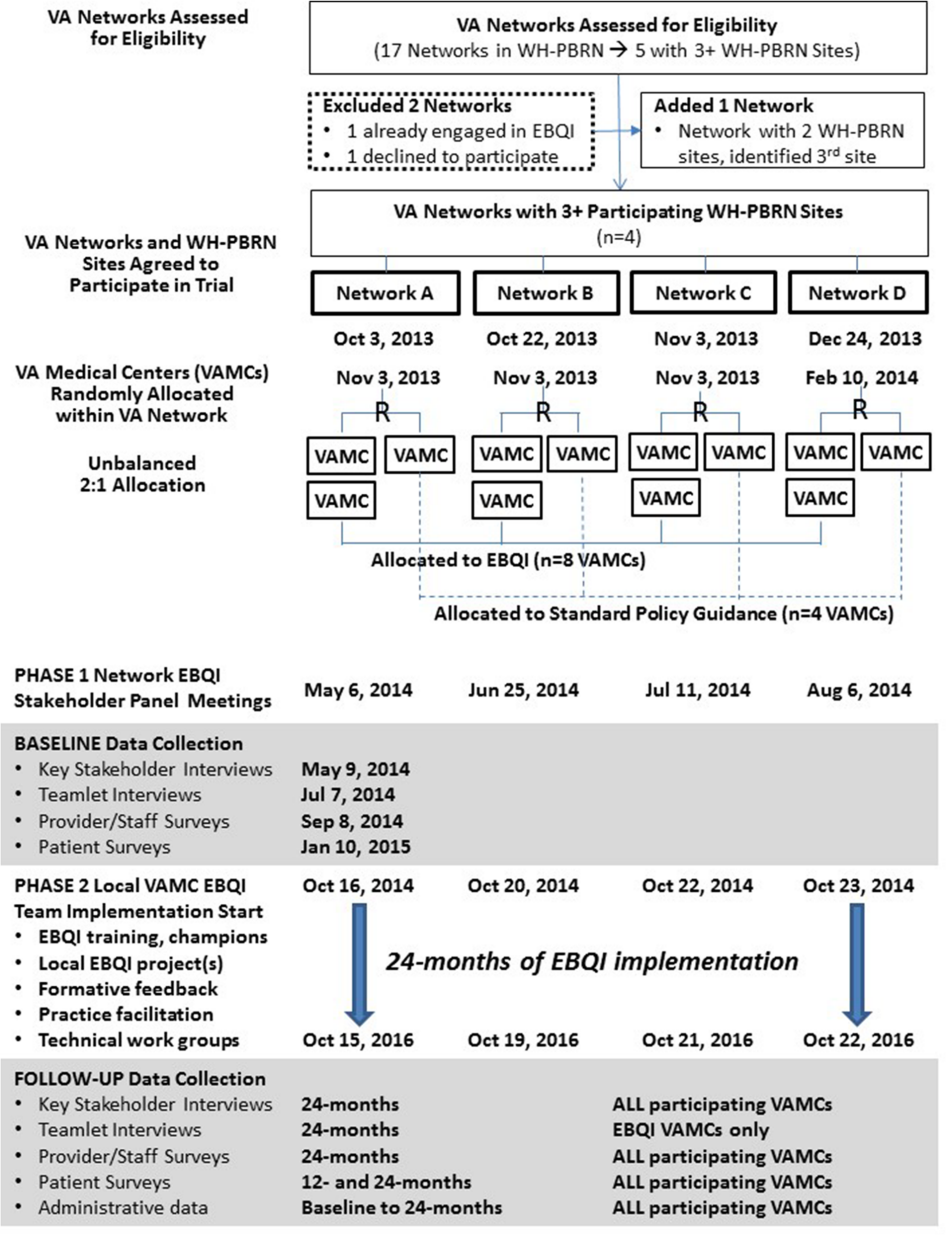
CONSORT flow diagram for cluster randomized controlled trial of evidence-based quality improvement

This study is designed as a parallel two-arm, cluster randomized controlled trial (cRCT), blocked on VISN (Fig. 1). We randomly assigned the 12 VAMCs to EBQI or usual PACT implementation in an unbalanced 2:1 ratio within VISN, supporting appraisal of variations in EBQI implementation in the context of differences of VISN geography, resources, and oversight (Fig. 1). No site stratification or matching criteria were used. The study biostatistician used www.randomization.com with a randomized permuted block of three (simple block of three VAMCs within each VISN) and a seed of 15,356 to start the random allocation sequence. The eight intervention VAMCs will engage in EBQI, while the four control VAMCs will receive standard PACT and WH care delivery handbooks and guidance that all VA facilities receive. The study biostatistician assigned the VAMCs to EBQI or control, while the study PI (EMY) enrolled and launched EBQI with the resulting eight VAMCs.

Local quality improvement (QI) teams, their WH and PC leaders, and VAMC facility leadership, as well as VISN leaders and other key stakeholders (e.g., national VA partners), will be the focus of this study, given their varying levels of responsibility for WH, primary care, and PACT implementation. Outcomes for the PC providers and WVs seen in each participating VAMC’s primary care/PACT programs, whether sited in a women’s clinic or general primary care clinic, will also be examined.

### Ethical approval and informed consent

The VA supports a large number of Institutional Review Boards (IRBs) across the USA among chiefly academically affiliated VAMCs, in addition to a Central IRB (cIRB) for multisite research. While this study represents a 12-VAMC cluster randomized trial, the cIRB determined that participating sites were not engaged in research (i.e., the intervention is QI, and all research activities are performed centrally at the Principal Investigator’s site in the VA Greater Los Angeles (GLA) Healthcare System), permitting the use of the local GLA IRB. Evaluation and data collection activities are submitted serially to the GLA IRB for review as discrete human subject research component projects (e.g., patient survey component, provider/staff survey component). Two components involved contractors at local affiliates (teamlet interviews with UCLA, provider/staff survey with RAND Health), whose IRBs also reviewed and approved their respective activities.

The study is registered with ClinicalTrials.gov (NCT02039856).

### VA partnerships

This study is one of the five inter-related projects that together comprise a WH-focused research-clinical partnership funded by VA Health Services Research and Development (HSR&D) Service under the Collaborative Research to Enhance and Advance Transformation and Excellence (CREATE) initiative [18]. Central to CREATE is the involvement of policy and/or practice partners in the design, conduct, and dissemination of study results, with an emphasis on implementation and spread of successful strategies. Within the VA Office of Patient Care Services, Women’s Health Services (WHS) is the WH CREATE’s primary partner, with close involvement of Mental Health Services (MHS) in assessing within- and across-project mental health analyses and themes. The WH CREATE is also advised by an Executive Steering Committee, comprised of WHS and MHS partners, representatives in national VA clinical quality reporting, regional network leadership, public affairs, WH clinical care delivery, WH policy, and implementation science and economics, as well as WVs who use the VA for care.

### Conceptual framework for application of EBQI

This study is guided conceptually and practically by the Chronic Care Model (CCM) [19]. Developed more than a decade ago, the CCM has been widely adopted to help guide clinical QI initiatives in the context of practices or teams in the USA and abroad [20]. The CCM depicts the health system linked with complementary community resources, while providers and teams within each healthcare organization (regardless of size) aim to deliver care that is characterized by consistent, evidence-based assessment, treatment, and follow-up, with clinical decision support (often via information technology) and support for patient self-management [21]. Applied to EBQI, local QI teams work on improvement plans that consider one or more CCM elements.

The CCM is a particularly strong fit for QI around medical homes, with an easy crosswalk to PACT goals. For example, for care coordination, PACT teamlets should link patients with community resources to facilitate referrals and respond to social service needs; provide care management services for high-risk patients; integrate behavioral health and specialty care through structured collaboration, co-location, or referral protocols; track and support patients when they obtain services outside the practice; follow up with patients within a few days of an emergency room visit or hospital discharge; and communicate test results and care plans to patients/families [22].

Addressing CCM elements, however, requires substantial stakeholder buy-in, local knowledge and skills in QI, availability of needed technical support, and continual guidance. Previous research has shown that evidence-based programs require adaptation to organizational values, needs, and resources prior to dissemination [23]. More structured than continuous quality improvement (CQI), which has had mixed results, EBQI is a systematic approach to developing a multilevel research-clinical partnership approach to QI, using top-down/bottom-up features to engage senior organizational leaders and local QI teams in implementing improvements in the context of prior evidence, provider behavior change methods, and local practice structure and resources [24]. National strategic directives serve as guides, while regional expert panels set innovation design priorities [25]. Local interdisciplinary QI teams design and implement local activities, while researchers serve as technical experts and guides. EBQI also uses team-based CQI methods to help teams structure their aims and measures and conduct plan-do-study-act (PDSA) cycles, in addition to convening topic-focused workgroups with research/clinical expertise with periodic across-site meetings for training and sharing data and lessons learned. EBQI’s value-added contribution is an emphasis on (a) applying objective evidence, with (b) theory review and synthesis integrated into aspects of innovation design and implementation, (c) valid and reliable measurement, and (d) formal measurement feedback to stakeholders at all levels [26]. Effectively applied to a series of VA implementation studies [27–29], EBQI uses well-established implementation strategies, such as local priority setting among key stakeholders, adaptation of the evidence to local context (practice tailoring), audit-and-feedback of QI data to support rapid cycle improvements, and practice facilitation to support implementation into practice [30].

### EBQI implementation strategies applied to WH-PACT and hypotheses

**Fig. 2:**
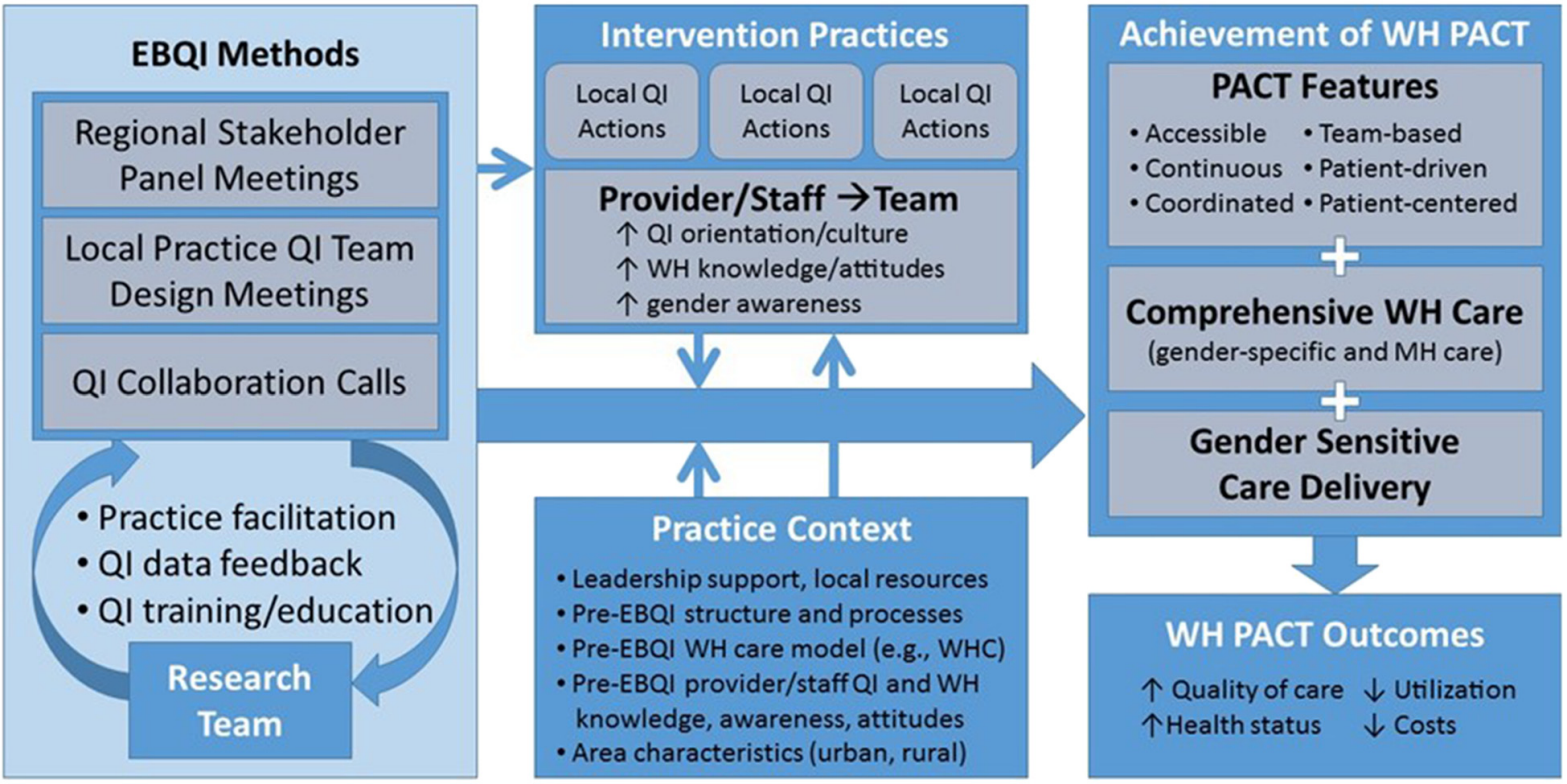
Evidence-based quality improvement (EBQI) conceptual model

In this study, we propose to use EBQI to convene multilevel stakeholder panels (presenting panels with evidence on factors associated with improved WH care in the context of national VA WH policy); facilitate local practice QI team design meetings, while providing QI training/education and iterative QI data feedback; and sponsor within and across-practice QI collaboration calls (Fig. 2). We will continually foster coverage of CCM elements.

We hypothesize that the initial results of EBQI will be the specification of the design choices for WH-PACT implementation, conduct of local QI activities, and improved provider/staff (and team) QI orientation, WH knowledge and attitudes, and gender awareness. We further hypothesize that EBQI will lead to higher achievement of WH-PACT (more practices achieve more features) in EBQI vs. control practices (aim #1). We anticipate that practice context (e.g., local leadership support, pre-EBQI factors) will moderate the relationships between EBQI and implementation of WH-PACT. We will then explore the extent to which receipt of WH-PACT concordant care (PACT features, comprehensive WH care, gender sensitive care delivery) is associated with improved WH-PACT outcomes (aim #2).

For aim #3 (processes), we will document EBQI activities among intervention practices and examine barriers and facilitators to WH-PACT achievement in all participating practices. For aim #3, we will also rely on Diffusion of Innovation theory [31, 32] to frame our assessment of EBQI-supported WH-PACT adaptation, implementation, and sustainability, considering, for example, the trialability, complexity, and relative advantage of EBQI-supported WH-PACT, as well as concordance between WH-PACT achievement and CCM principles. Aim #4 (tools) will build on our conceptual model and benefit from our practical experience implementing evidence-based care models [15].

In this study, EBQI implementation will focus on six main activities: (1) conduct of four VISN-level interdisciplinary stakeholder planning meetings using expert panel techniques to come to consensus on PACT QI priorities for women Veterans (“QI roadmaps”); (2) development and training of a local QI champion and QI team members at EBQI-assigned VAMCs to pursue one or more QI projects from the “roadmaps”; (3) formative feedback from patient, provider, staff, and practice survey data; key stakeholder and teamlet interview data; and utilization and cost-related administrative data; (4) ongoing practice facilitation and expert review and feedback on local QI proposals and progress; (5) monthly across-intervention VAMC calls to facilitate collaboration and spread of effective QI innovations; and (6) technical work groups designed to provide additional evidence-based support in priority areas (Table 1).

**Table 1:** Core components of evidence-based quality improvement (EBQI) implementation strategy

EBQI component	Activities	Example product(s)
**Conduct of VISN-level interdisciplinary stakeholder planning meetings to develop “QI roadmaps”**	Modified Delphi panel meetings with materials on PACT and panel ratings in advance of an in-person presentation of aggregated pre-panel ratings for review and moderated discussion and consensus development on top priorities for QI in context of feasibility of implementation and local resources	**Panel materials** • Panel rating form • Summary of women Veterans’ research • VISN-level gender differences in patient ratings of care • Local practice and patient population characteristics **VISN-level QI roadmaps** • Brief panel presentation summary • Brief summary of top VISN priorities for QI for PACT for women Veterans • Brief summary of top-rated topics requiring technical support (e.g., care coordination between VA and non-VA providers) • Oversight and communication plans
**Development, training, and support of local QI champions and QI team members**	In-person training of 1–2 local QI champions at the parent study site in Los Angeles • National PC and WH leadership endorsement • Review VISN QI roadmaps and planned QI projects across intervention sites • Review approaches to QI • Breakout groups on applying QI methods/tools to project plans • Across-team debriefing • Formative feedback from key stakeholder interviews • Breakout groups on applying EBQI principles to WH-PACT QI projects • Training on EBQI formative feedback reports and Technical Work Groups • Exemplar session on using EBQI to improve PACT team functioning • Q&A panel with EBQI experts on lessons learned from prior projects • Training on local QI project documentation	**Training materials** (reader, slidesets, breakout exercises, in-person expert EBQI project consultations) **Follow-up technical consults on local QI plans** (with QI/system redesign consultant by email/phone)
**Formative feedback of local QI data**	• Feedback of baseline and 12-month survey data from women Veterans seen in participating VAMC primary care clinics • Feedback of baseline PACT provider and staff survey findings • Feedback of key themes from baseline interviews of VISN, VAMC and practice-level key stakeholders • Feedback of key themes from baseline interviews of PACT teamlet members in participating VAMCs • Feedback of VA quality measures and patient survey data by gender for participating VAMCs	**Site-level formative feedback reports** with comparisons to VISN and all participating VAMCs
**Ongoing practice facilitation and expert review/feedback on local QI proposals and progress**	• Regular EBQI team contacts with local QI teams by telephone and email • Troubleshooting of local problems using VISN oversight/communication plans • Intermittent policy contacts (e.g., identify/disseminate key policy documents, obtain national guidance)	**Structured local QI project proposals** (templated) **Structured expert feedback** (email and telephone summaries)
**Facilitation or across-site collaboration and spread of effective QI innovations**	EBQI team-moderated monthly calls with 1+ representative per intervention VAMC	**Verbal summaries of local QI project progress** (including shared materials across sites) **Aggregated across-site formative feedback** (from multiple data sources, e.g., patient surveys)
**Technical work groups designed to provide additional evidence-based support in priority areas**	VISN-level stakeholder panel meeting (above) used to also rate priority areas in which expert evidence-based consultation and support would be useful—work groups will be convened among national experts in clinical care and research in selected priority areas	**Mini-systematic reviews** in priority area(s) **Practice scans** of WH-PBRN site leads to identify best practices at other VAMCs

### Evaluation

**Fig. 3:**
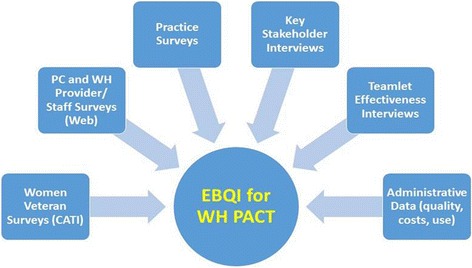
Evaluation components of the Women’s Health PACT trial. Legend: *PC* primary care, *WH* women’s health, *CATI* computer-assisted telephone interviews

As shown in Fig. 3, we have planned a comprehensive approach to evaluating the processes and outcomes of EBQI for tailoring PACT to the needs of WVs. Baseline data collection (as well as 12-month follow-up in the case of the patient surveys) will be used for formative feedback to EBQI-assigned VAMCs. Table 2 provides an overview of the data sources, samples, and measures planned for each evaluation component.

**Table 2:** Formative and summative evaluation data sources, samples, and measures

Data sources and samples	Measures
**Women Veteran patient surveys** (baseline, 12-month and 24-month follow-up)	
Random samples of complete enumerated list of women Veterans age 18 or older enrolled as VA patients with at least 3 primary care or women’s health clinic visits in the 12 months prior to the start of the baseline survey (target final enrollment of 40 women Veterans at each site for total of 480 at all 12 participating sites by 24-month follow-up)	• Healthcare utilization (VA, non-VA, dual use, VA-paid community care) • Overall VA, VA primary care, women’s health, mental health use and quality ratings, satisfaction • Trauma-sensitive primary care (exams, provider communication around trauma histories) • Preferences for care (e.g., same-gender provider, women’s clinic) • Access to care (e.g., appointments, waiting rooms, phone access, including barriers, delays, missed care) • Continuity of care • Mental health needs (anxiety, depression, posttraumatic stress disorder screens) • Care coordination (e.g., with specialists, non-VA) • Comprehensiveness of care (e.g., available services) • Provider-patient communication • Gender sensitivity • Psychosocial safety • Trust in providers • Health status, functional status, comorbidities • Military exposures (e.g., combat, harassment) • Lifetime trauma exposure • Sociodemographics
**VA provider and staff surveys** (baseline and 24-month follow-up)	
Census of all VA providers and staff who hold positions as non-resident providers (MDs, NPs, PAs), nurses (RNs, LVNs, LPNs), administrative staff (clerks, medical support assistants), or PACT greater team professionals (e.g., social workers, pharmacists, dieticians, health educators, health coaches, and co-located mental health providers) who practice or work at one of the 12 participating sites and who are members of their local general PC/PACT or WH-PACT teams	• EBQI exposure/participation (e.g., # hours spent, QI training time, awareness/knowledge, barriers/facilitators) • QI orientation/culture (e.g., participative decision-making, readiness to change) • Gender awareness (e.g., gender sensitivity, knowledge, women’s health knowledge/attitudes, self-assessment of proficiency) • PACT achievement (e.g., team-based care, teamlet communication, knowledge and skills, psychological safety, openness to new ideas, ease/difficulty of integrating women Veterans’ preferences and needs) • Practice context (e.g., leadership norms, organizational readiness to change, job satisfaction) • Provider/staff characteristics (e.g., age, gender, race, ethnicity, clinician type, designated women’s health provider, panel characteristics, half-days in clinic)
**VA practice surveys** (baseline and 24-month follow-up)	
Primary care and women’s health clinic leaders (all 12 participating sites)	• Leadership support (e.g., degree of buy-in, leadership involvement in EBQI) • Local resources (e.g., sufficiency of resources to implement PACT, meet comprehensive care needs) • Practice structure (e.g., type of clinic model, separate vs. integrated clinic space, onsite vs. offsite services) • Ability to engage in EBQI (e.g., QI orientation in PC, in WH, barriers to local QI, practice stress) • Gender-sensitive care environment (e.g., availability of same-gender providers, privacy, sufficiency of equipment for pelvic exams) • Access to gender-specific care (e.g., availability of designated women’s health providers, gynecologists; reliance on VA vs. community care) • PACT implementation (e.g., teamlet staffing ratios, teamlet function, secure messaging) • Practice characteristics (e.g., practice size, urban or rural location, academic affiliation, years in operation)
**Key stakeholder interviews** (baseline and 24-month follow-up)	
Purposive sample of 48 or more VA primary care directors, women’s health medical directors, Women Veteran Program Managers, and VA medical center leadership (all 12 participating sites)	• EBQI activities (e.g., site initiation of EBQI activities, leadership and stakeholder/staff involvement) • PACT implementation (e.g., general PACT and women’s health PACT activities and implementation issues) • Facilitators/barriers to implementation of PACT in general and for women Veterans specifically (e.g., sufficiency of resources/time, training needed)
**PACT teamlet interviews** (baseline and 24-month follow-up)	
Stratified random sample of 36 or more PACT teamlet members stratified by role (8 EBQI sites only): • Non-resident providers (MD, NP, PA) • Nurses (RN, LVN, LPN) • Administrative staff (clerk, medical support assistant) • Co-located mental health professionals who practice or work in the general PC/PACT and/or WH-PACT teams	• Teamlet composition and roles (e.g., members, formation, task allocation) • Teamlet structured communication (e.g., meetings, use of huddles, training, coordinating care with specialists) • PACT teamlet and practice changes (e.g., role expansions, performance feedback) • Access/continuity of care (e.g., improvements, non-face-to-face care, reducing/preventing walk-ins, group visits) • Impact of PACT changes (e.g., planning and implementing changes, small tests of change, resources needed, leadership support, teamlet reactions) • Communicating strategies for improvement (e.g., how teamlets improve together, how best practices are shared)
**Administrative data** (retrospective quarterly data pulls over 24-month period)	
Area-, organizational (VA medical center and clinic-level)-, provider- and patient-level data (all 12 participating sites)	• Quality of care measures from VA performance measures (chart-based and patient-survey-based measures by gender), including prevention and chronic disease metrics (e.g., immunizations, cancer screening, diabetes process measures) and patient ratings of access, continuity and coordination • Utilization and cost measures (e.g., total annual costs per patient and utilization by type of care, panel sizes) • Organizational measures (e.g., facility complexity) • Provider characteristics (e.g., primary care and women’s health provider types, volume, ratios) • Patient-level measures (e.g., primary care, women’s health, mental health, specialty care visits, hospitalizations, emergency room visits; diagnoses)

*MDs* medical doctors, *NPs* nurse practitioners, *PAs* physician assistants, *RNs* registered nurses, *LVNs* licensed vocational nurses, *LPNs* licensed practical nurses, *PC* primary care, *PACT* Patient Aligned Care Teams, *WH* women’s health, *VISN* Veterans Integrated Service Network

### Sample size calculation and power analysis

We based our power calculations on the study’s unequal (2:1) intervention-to-control ratio and clustered sample design. To detect a moderate (0.20) effect size between WVs at intervention and control sites, we considered data from the Primary Care Satisfaction Survey for Women’s Care Coordination and Comprehensiveness scale, [33] with 12 practices (8 intervention-to-4 control sites), cluster adjustment (ICC = 0.03) [34], and two-tailed 5 % significance level. Based on these parameters, we will target enrollment of a minimum of 40 WVs at each of the 12 study sites over the 2-year period (baseline to 24-month follow-up), for a total of 480 WVs who complete a baseline and at least one of the two follow-up surveys (12- and/or 24-month). To achieve this target, we will create a baseline sample of 6665 WVs who have received PC or WH care at one of the study’s 12 participating sites during the 12-month period prior to the baseline survey (555 per site). Allowing for a conservative 20 % response rate and 40 % attrition over the 2-year period, we aim to enroll 111 WVs at each of the 12 sites at baseline, for a total of 1333 interviews at the completion of the first wave.

### Primary outcome measures

The primary outcome measure, focused on achievement of the PACT model of care for WVs, will include changes in levels of achievement of individual PACT features: access, continuity, care coordination, team-based care, patient-driven care, patient-centeredness, comprehensiveness (including gender-specific services and integrated MH), and gender sensitivity (Fig. 2). These measures will be examined at the practice, provider/staff, and patient levels and investigated qualitatively using the key stakeholder and teamlet interviews.

### Secondary outcome measures

Secondary outcome measures include WH-PACT outcomes that should result from achievement of the full PACT model, including improvements in quality of care and health status, and reductions in utilization and costs. For quality of care, we will examine chronic disease quality (e.g., process measures such as foot sensation exams or eye exams among diabetics) and preventive practice (e.g., women’s breast and cervical cancer screening and gender-neutral prevention, such as influenza immunizations and colorectal cancer screening). We will determine whether total annual costs per patient and utilization by type of care (e.g., primary care, specialty care, hospitalizations, emergency department visit rates) have changed in EBQI vs. routine PACT implementation VAMCs and based on level of WH-PACT achievement. We anticipate, however, that acute and emergency care may be too rare in our practice populations to have the power to detect differences over time, so these analyses will be exploratory.

We will also examine impacts on provider and staff WH knowledge and attitudes, QI orientation/culture, and gender awareness, as well as changes in practice contextual factors as a result of EBQI exposure (e.g., changes in leadership support, local resources applied to WH and primary care QI, training).

### Statistical analyses

We will use multivariate regression to determine EBQI effectiveness, adjusting for covariates, clustering, and enrollment and attrition weights at the patient and provider/staff levels. The primary regressor of interest is being at an EBQI vs. routine PACT implementation site. We will examine the potential moderating effects of practice context and provider/staff knowledge/attitudes. Patient-level controls will include sociodemographic characteristics, health status, comorbidity, and utilization. Adjustment for clustering will be performed using Stata v13. We will evaluate goodness-of-fit using Mallow’s statistic (C_p_). We will use multiple imputation methods to address missing data patterns among covariates, although CATI procedures will significantly reduce item non-response in the patient survey, and the web-survey methods will do the same for the provider/staff surveys. Hot deck imputation will be used for imputation of missing values within scales as needed. To address the potential for response bias, patient and provider/staff survey data will be weighted to the inverse of the probability of selection based on available characteristics in the administrative data used to randomly sample them. We will use factor analysis and cluster analysis using PACT features (e.g., access), comprehensive women’s health care (e.g., gender-specific care availability), and gender-sensitive care delivery (e.g., availability of same-gender providers or gender-aware providers) for WH-PACT achievement variable creation.

All semi-structured interviews will be audio-recorded and transcribed, and qualitative analysis will be conducted using ATLAS.ti version 7. Interview domains are noted in Table 2. Initially, a top-level codebook will be developed for the baseline interviews based on the interview guide. The codebook will then be elaborated based on emergent themes using a constant comparison analytic approach, adjusting as each round of interviews is reviewed. Interviews will be compared within and across facilities and over time. In baseline key stakeholder and teamlet transcripts, we will conduct targeted coding of PACT-related knowledge, attitudes, beliefs, and experiences; expectations of WH-PACT’s effectiveness; and practice contextual factors that influence how care is delivered (and changed) locally. In follow-up key stakeholder transcripts, we will identify factors that facilitated and/or impeded EBQI and WH-PACT achievement and strengths/weaknesses of the WH-PACT model as implemented.

### Trial timeframe

The WH-PACT trial is planned from March 2013 through February 2017 (Fig. 1). Phase 1 network-level stakeholder panel meetings occurred as half-day in-person meetings from May to August 2014. Phase 2 local QI launched at all eight EBQI-assigned VAMCs in October 2014. Baseline key stakeholder interviews in all participating VISN and VAMC followed within days of each respective network-level meeting, followed by teamlet interviews at EBQI sites only within one quarter of each meeting. Provider and staff surveys were fielded to PC and WH providers and staff in all participating VAMCs starting September 2014, while baseline patient surveys were launched in January 2015, yielding formative feedback in the following year. EBQI will run for 24 months from launch of local QI teams, followed by 24-month follow-up interview and survey waves.

### Trial status

Baseline data collection and EBQI implementation phase.

## Discussion

This trial represents an important opportunity to tailor VA’s PCMH to the needs of WVs while also providing new implementation science insights. This study is one of the first of its kind to test EBQI in the context of the VA WH-PBRN, building on lessons learned from a pilot EBQI study in the founder sites conducted in the first few years of PBRN implementation [35]. The value-added of PBRN use for sustainable practice-based changes in VA settings adds another dimension to this work. We anticipate that we will glean important new knowledge of what is needed to accomplish multilevel stakeholder engagement in evidence-based organizational changes.

The VA has undergone significant changes in the past 30 years since the Government Accounting Office first identified deficits in care for women in 1982 [1]. There is a growing awareness of the vulnerability of women in the military, [36, 37] with substantial MH burdens, [38] and increasing demands in an array of new mandated WH services (such as maternity benefits and newborn care), all occurring in a healthcare system with a characteristically low volume of women. While VA managers have economic incentives to increase market share among women, an underlying tension remains unresolved on how best to achieve the tenets of gender-sensitive comprehensive WH care [39, 40].

We anticipate that study results will demonstrate the value of EBQI approaches to adapting PACT to the needs of special populations, ensuring PACT not only meets the needs of WVs but also serves as an exemplar for other special populations (e.g., recently returned veterans who may need specific post-deployment health care, veterans with HIV who sometimes obtain PC in infectious disease clinics). Given the growing number of WVs relying on VA care, the expansion of services for which WVs are now eligible [41], and the pressing need to reduce persistent gender disparities in VA care, the VA will reap important benefits from determining how to adapt PACT to enhance WVs’ outcomes. And, by systematically engaging frontline leaders, managers, and providers, our approach will substantially increase the relevance and thereby potential sustainability and spread of the resulting WH-PACT model.

In this study, we will develop and test methods for providing the kind of organizational supports that the Institute of Medicine’s *Crossing the Quality Chasm* referred to as central to transformative change [42]. This study reflects the first time these methods will be deployed to address the needs of WVs, tied to careful, comprehensive measurement and evaluation, which in turn will inform an evidence-based roadmap for policy/practice action and a foundation for developing evidence-based tools to support scale-up and spread to different VA types in different contexts. The strength of our partnerships with WH and primary care leadership will also ensure that we are continually grounded in fiscal, management, and strategic issues of consequence as the study is conducted. Tackling the fundamental tenets of PACT care in gender-sensitive and patient-centered ways will clearly be essential to retaining WVs in care and fulfilling the promise to ensure that WVs get “the best care anywhere” [43].

## Abbreviations

CCM, Chronic Care Model; CQI, continuous quality improvement; EBQI, evidence-based quality improvement; ICC, intraclass correlation coefficient; LPN, licensed practical nurse; LVN, licensed vocational nurse; MD, medical doctor; MH, mental health; NP, nurse practitioner; PA, physician’s assistant; PACT, Patient Aligned Care Teams; PBRN, practice based research network; PC, primary care; PCMH, patient-centered medical homes; PDSA, Plan, Do, Study, Act; QI, quality improvement; RN, registered nurse; VA, US Department of Veterans Affairs or Veterans Health Administration healthcare system; VAMC, VA medical center; VISN, Veterans Integrated Service Network; WH, women’s health; WHS, Women’s Health Services; WVs, women Veterans

## References

[1] U.S. General Accounting Office (1982). Actions needed to ensure that female veterans have equal access to VA benefits.

[2] Veterans Health Administration (2010). Sourcebook: women veterans in the Veterans Health Administration. Volume 1: sociodemographic characteristics and use of VHA care.

[3] Yano EM, Rose D, Bean-Mayberry B, Canelo I, Washington DL (2010). Impact of practice structure on the quality of care for women veterans (phase 2) final report.

[4] Veterans Health Administration (2008). Report to the Appropriations Committee of the U.S. House of Representatives in response to House Appropriations Report No. 110–186, accompanying Public Law 110–161, The Consolidated Appropriations Act.

[5] Vogt DS, Stone ER, Salgado DM, King LA, King DW, Savarese VW (2001). Gender awareness among veterans administration health-care workers: existing strengths and areas for improvement. Women’s Health.

[6] Murdoch M, Bradley A, Mather S, Klein R, Turner C, Yano EM (2006). Women and war: what physicians should know. J Gen Intern Med..

[7] Starfield B, Shi L, Macinko J (2005). Contribution of primary care to health systems and health. Milbank Q..

[8] Starfield B, Shi L (2004). The medical home, access to care, and insurance: a review of evidence. Pediatrics..

[9] Klein S (2011). The Veterans Health Administration: implementing patient centered medical homes in the nation’s largest integrated delivery system. Commonw Fund..

[10] Veterans Health Administration (2010). Health care services for women veterans. VHA Handbook 1330.01.

[11] Yano EM, Haskell S, Hayes P (2014). Delivery of gender-sensitive comprehensive primary care for women veterans: implications for VA patient aligned care teams. J Gen Intern Med.

[12] Veterans Health Administration (2012). Gender differences in performance measures, VHA 2008–2011.

[13] Wagner EH, Austin BT, Davis C, Hindmarsh M, Schaefer J, Bonomi A (2001). Improving chronic illness care: translating evidence into action. Health Affairs.

[14] Glasgow RE, Orleans CT, Wagner EH (2001). Does the chronic care model serve also as a template for improving prevention?. Milbank Q.

[15] Rubenstein LV, Stockdale SE, Sapir N, Altman L, Dresselhaus T, Salem-Schatz S, Vivell S, Ovretveit J, Hamilton AB, Yano EM (2014). A patient-centered primary care practice approach using evidence-based quality improvement: rationale, methods, and early assessment of implementation. J Gen Intern Med.

[16] Yano EM, Yih Y (2010). The VA health care delivery system. Handbook of healthcare delivery systems.

[17] Frayne SM, Carney DV, Bastian L, Bean-Mayberry B, Sadler AN, Klap R (2013). The VA women’s health practice-based research network: amplifying women veterans’ voices in VA research. J Gen Intern Med..

[18] Yano EM (2015). A partnered research initiative to accelerate implementation of comprehensive care for women veterans: the VA women’s health CREATE. Med Care.

[19] Bodenheimer T, Wagner EH, Grumbach K (2002). Improving primary care for patients with chronic illness. JAMA.

[20] Coleman K, Austin BT, Brach C, Wagner EH (2009). Evidence on the chronic care model in the new millennium. Health Aff (Millwood).

[21] Bodenheimer T, Wagner EH, Grumbach K (2002). Improving primary care for patients with chronic illness: the chronic care model, part 2. JAMA.

[22] The Chronic Care Model and Patient Centered Medical Homes. Improving Chronic Illness Care. http://www.improvingchroniccare.org/index.php?p=Care_Coordination&s=258. Accessed 10 May 2016.

[23] Gold PB, Glynn SM, Mueser KT (2006). Challenges to implementing and sustaining comprehensive mental health service programs. Eval Health Prof..

[24] Parker LE, dePillis E, Altschuler A, Rubenstein LV, Meredith LS (2007). Balancing participation and expertise: a comparison of locally and centrally managed health care quality improvement within primary care practices. Qual Health Res.

[25] Rubenstein LV, Fink A, Yano EM, Simon B, Chernof B, Robbins AS (1995). Increasing the impact of quality improvement on health: an expert panel method for setting institutional priorities. Jt Comm J Qual Improve..

[26] Rubenstein LV, Meredith LS, Parker LE, Gordon NP, Hickey SC, Oken C, Lee ML (2006). Impacts of evidence-based quality improvement on depression in primary care: a randomized experiment. J Gen Intern Med.

[27] Chaney EF, Rubenstein LV, Liu C-F, Yano EM, Bolkan C, Lee ML (2011). Implementing collaborative care for depression treatment in primary care: a cluster randomized evaluation of a quality improvement practice redesign. Implement Sci..

[28] Yano EM, Rubenstein LV, Farmer MM, Chernof BA, Mittman BS, Lanto AB (2008). Targeting primary care referrals to smoking cessation clinics does not improve quit rates: implementing evidence-based interventions into practice. Health Serv Res..

[29] Brown AH, Cohen AN, Chinman MJ, Kessler C, Young AS (2008). EQUIP: implementing chronic care principles and applying formative evaluation methods to improve schizophrenia. Implement Sci..

[30] Rubenstein LV, Chaney EF, Ober S, Felker B, Sherman SE, Lanto A, Vivell S (2011). Using evidence-based quality improvement methods for translating depression collaborative care research into practice. Fam Syst Health.

[31] Rogers EM (1995). Diffusion of innovation.

[32] Greenhalgh T, Robert G, Macfarlane F, Bate P, Kyriakidou O (2004). Diffusion of innovations in service organizations: systematic review and recommendations. Milbank Q.

[33] Scholle SH, Weisman CS, Anderson R, Weitz T, Freund KM, Binko J (2000). Women’s satisfaction with primary care: a new measurement effort from the PHS national centers of excellence in women’s health. Women’s Health Issues.

[34] Adams G, Gulliford MC, Ukoumunne OC, Eldridge S, Chinn S, Campbell MJ (2004). Patterns of intra-cluster correlation from primary care research to inform study design and analysis. J Clin Epidemiol.

[35] Fox AB, Hamilton AB, Frayne SM, Wiltsey-Stirman S, Bean-Mayberry B, Carney D, DiLeone B, Giersch JM, Goldstein KM, Romodan Y, Sadler A, Wiltsey-Stirman S, Yano EM, Yee E, Vogt D. Effectiveness of an evidence-based quality improvement approach to cultural competence training: The Veterans Affairs’ “Caring for Women Veterans” program. J Contin Educ Health Prof. 2016; [in press]. 10.1097/CEH.0000000000000073 PMC8082471 27262152

[36] Suffoletta-Maierle S, Grubaugh AL, Magruder K, Monnier J, Frueh BC (2003). Trauma-related mental health needs and service utilization among female veterans. J Psychiatr Pract.

[37] Murdoch M, Polusny MA, Hodges J, O'Brien N (2004). Prevalence of in-service and post-service sexual assault among combat and noncombat veterans applying for VA PTSD disability benefits. Mil Med.

[38] Magruder KM, Frueh BC, Knapp RG, Johnson MR, Vaughan JA, Carson TC (2004). PTSD symptoms, demographic characteristics, and functional status among veterans treated in VA primary care clinics. J Trauma Stress.

[39] Weisman CC, Curbow B, Khoury AJ (1996). Women’s health centers and managed care. Women’s Health Issues..

[40] deKleijn M, Lagro-Janssen TA, Canelo I, Yano EM (2015). Creating a roadmap for delivering gender-sensitive comprehensive care for women Veterans: results of a national expert panel. Med Care.

[41] Washington DL, Caffrey C, Goldzweig C, Simon B, Yano EM (2003). Availability of comprehensive women’s health care through Veterans Affairs Medical Centers. Women’s Health Issues..

[42] Institute of Medicine (2004). Crossing the quality chasm.

[43] Longman P. The best care anywhere. Washington Monthly. 2005. http://www.ssc.wisc.edu/soc/faculty/pages/wright/longman%20paper%20for%20soc125.html. Accessed 10 May 2016

